# Perceptive Dialogue for Linking Stakeholders and Units During Care Transitions – A Qualitative Study of People with Stroke, Significant Others and Healthcare Professionals in Sweden

**DOI:** 10.5334/ijic.4689

**Published:** 2020-03-25

**Authors:** Sebastian Lindblom, Charlotte Ytterberg, Marie Elf, Maria Flink

**Affiliations:** 1 Division of Physiotherapy, Department of Neurobiology, Care Sciences and Society, Karolinska Institutet, Huddinge, SE; 2 Function Area Occupational Therapy and Physiotherapy, Allied Health Professionals, Karolinska University Hospital, Stockholm, SE; 3 School of Education, Health and Social Studies, Dalarna University, Falun, SE; 4 Division of Family Medicine and Primary Care, Department of Neurobiology, Care Sciences and Society, Karolinska Institutet, Huddinge, SE; 5 Function Area Social Work in Healthcare, Allied Health Professionals, Karolinska University Hospital, Stockholm, SE

**Keywords:** transitional care, care coordination, continuity of care, patient handoff, rehabilitation

## Abstract

**Introduction:**

Care transitions are a complex set of actions that risk poor quality outcomes for patients and their significant others. This study explored the transition process between hospital and continued rehabilitation in the home. The process is explored from the perspectives of people with stroke, significant others and healthcare professionals in Stockholm, Sweden.

**Method:**

Focus group interviews (n = 10), semi-structured individual interviews (n = 23) and interviews in dyad (n = 4) were conducted with healthcare professionals, people with stroke and significant others, altogether 71 participants. Data was collected and analyzed using Grounded Theory.

**Results:**

One core category *“Perceptive dialogue for a coordinated transition”, and two categories “Synthesis of parallel processes for common understanding”* and *“The forced transformation from passive attendant to uninformed agent”* emerged from the analysis. The transition consisted of several parallel processes which made it difficult for the stakeholders to get a common understanding of the transition as a whole. Enabling a perceptive dialogue was as a prerequisite for the creation of a common understanding of the care transition.

**Conclusion:**

This study elucidates that a perceptive dialogue with patients/significant others as well as within and across organizations is part of a coordinated and person-centred transition. There is an extensive need for increased involvement of patients and significant others regarding dialogue about health conditions, procedures at the hospital and preparation for self-management after discharge.

## Introduction

Since the beginning of the 21^st^ century, increased attention has been drawn towards the optimization of care transitions as an important part of the movement towards a more person-centred and integrated health care [1]. Care transitions, moving from one healthcare setting to another, have been identified as a vulnerable set of actions, with the potential risk of adverse events and re-hospitalization [2,3,4]. A lack of coordinated transitions can result in a substantial burden for patients and their families with a risk of information loss, unwanted outcomes and a lack of help in navigating the healthcare system [4,5,6,7].

Coordinated transitions are especially important for people with complex conditions who need care or rehabilitation from the various providers responsible, i.e. hospital, primary care and municipality. As the second leading cause of death and disability worldwide [8,9], stroke is one such complex condition with an abrupt onset and potentially life-changing consequences both for the patient and the significant others [10]. Despite an extensive literature on experiences of transitional care, the situation for people with stroke differs from those with other health conditions. Following the abrupt onset, people with stroke find themselves from one day to another transitioned to a sudden change of health condition with impairments and reduced capacity. This sudden change also means that they may lack both knowledge and experience on how to navigate the healthcare system. At the onset of stroke, immediate hospital care is required, and strong evidence supports that such care should be provided in specialized stroke units [11]. After the acute medical treatment, a subsequent need for post-discharge rehabilitation is most often required, e.g. discharge to in-patient rehabilitation or directly to the home with continued rehabilitation in the home environment [6]. Therefore, the discharge from the stroke unit typically entails a care transition from one healthcare provider to another, and the structure of the care pathway for people with stroke is often described as fragmented [12].

Early supported discharge (ESD) is a care transition model for people with stroke with strong evidence of beneficial outcomes [13,14]. The core component of the ESD is an interdisciplinary team that plans and coordinates the discharge together with the patient and continues the subsequent rehabilitation in the home environment. The team can either be situated at hospital and continue the hospital-initiated rehabilitation at home or be situated in primary care and start the rehabilitation at the hospital and continue at home. Despite the evidence [13,15] as well as policy documents and guidelines recommending the model [16,17,18], it has been challenges in the large-scale implementation [19,20,21]. It has been suggested that differences in stroke care organization hinders the application of proposed implementation guidelines [22]. As the ESD draws upon a need for professionals to work both in hospital and in the community extensive cross-organizational collaboration both on micro and macro level is needed. In systems with a split responsibility and organizational borders between levels of care, such cross-organizational movement is challenging.

Hence, people with stroke are a vulnerable group that struggle with sudden life-changing complications and most often lack experience of both their illness and the healthcare system. In healthcare systems without a large implementation of ESD such as in Sweden or the US [22], there is a need to understand how transitions can be coordinated across organizational borders and based on the needs of the individual patients.

Therefore, the aim of this study was to explore the transition process between hospital and the home with continued rehabilitation in the home environment. The process is explored from the perspectives of people with stroke, their significant others and healthcare professionals.

## Methods

### Study design

We conducted a qualitative study inspired by grounded theory as described by Charmaz [23].

### Settings and participants

The patients and healthcare professionals interviewed for this study were recruited from the acute stroke units and geriatric stroke units at two different hospitals and their corresponding primary care neuro-rehabilitation teams, all situated in Stockholm, Sweden. The hospitals were chosen according to their size and possibility to include unselected and mixed patients. Hospital A was a county hospital, situated within a prosperous socio-economic catchment area. Hospital B was a regional university hospital with a lower socio-economic catchment area.

Patients invited to participate in the study were people with stroke who were to be discharged from the acute or the geriatric stroke units to continued rehabilitation in the home environment. Significant others were identified by the patient with stroke and were asked to participate in the study. The neuro-rehabilitation teams consisted of occupational therapists, physiotherapists, speech and language therapists and social workers. The hospital healthcare professionals consisted of occupational therapists, physiotherapists, speech and language therapists, registered nurses and physicians.

All the participants received oral and written information about the study and informed consent was obtained. The Regional Ethics Committee in Stockholm approved the study.

### Data collection and analysis

Data was cyclical collected and analyzed from spring 2016 to early 2018. In total, 38 interviews of different types were conducted with altogether 71 participants: 10 focus groups interviews with 40 healthcare professionals from both hospital and primary care; 23 semi-structured individual interviews including 12 patients with stroke, 3 significant others, 1 stroke coordinator, 7 healthcare professionals; and 4 interviews in dyads constituting 4 patients with stroke and 4 significant others. Data on characteristics of the participants was collected from medical records and/or through interviews, see Table 1.

**Table 1:** Characteristics of participants.

People with stroke, n = 16	

Age, mean (SD) range	71 (11) 40–82
Sex, female/male, n	7/9
Cohabiting, yes/no, n	12/4
Working, yes/no, n	3/13
Length of stay at stroke unit, mean (SD) range	4 (4) 1–19
Length of stay at geriatric stroke unit, mean (SD) range	12 (10) 6–30
Length of stay total, mean, (SD) range	8 (12) 1–49
Stroke severity*	
Mild, n	10
Moderate/severe, n	6
**Significant others, n** = **7**	

Age, mean (SD) range	66 (10) 48–79
Sex, female/male, n	5/2
Cohabiting with person with stroke, yes/no, n	5/2
Working, yes/no, n	2/5
Relation to patient	
Wife/Husband, n	5
Daughter, n	1
Son, n	1
**Health care professionals, n** = **48**	

Age, mean (SD) range	44 (14) 25–70
Sex, female/male, n	44/4
Experience in occupation, years, mean (SD) range	15 (12) 0.5–43
Experience at current workplace, years, mean (SD) range	8 (8) 0.1–29
Occupation, n	
Physiotherapist	16
Occupational therapist	12
Physician	5
Nurse	6
Speech and language therapist	4
Social worker	3
Stroke coordinator	1
General practitioner	1
Location	
Acute stroke unit, n	19
Geriatric stroke unit, n	10
Neuro-rehabilitation team (primary care), n	19

* Stroke severity was assessed by the modified Rankin Scale.

Initially, data was only collected at Hospital A. For healthcare professionals, a purposive sampling was initially used to recruit professionals of different professions with positions that directly involved them in care transitions. The healthcare professionals were invited to participate during their day-time shifts at a for them convenient time and place. Those who were not able to participate during their shifts were offered other options to allow participation. For patients, purposive sampling was initially used to ensure a heterogeneous sample regarding age, gender, hospital unit, and stroke severity assessed by the modified Rankin Scale [24]. Through this, we aimed to collect as many perspectives of the care transition process as possible. As analysis progressed theoretical sampling was used [23]. During the theoretical sampling patients and healthcare professionals were included from hospital B. From the analysis of the initial 15 interviews, conducted with patients and professionals from hospital A and the related neuro-rehabilitation teams, an emerging category of trust and organizational routines was established. As this seemed to be related to the organization of the work environment, we concluded that in order to be able to check, refine and affirm the data, continued data collection was necessary within another context. A hospital with a different socioeconomic catchment area and a recent large re-organization of work structure and management, i.e. Hospital B, was therefore chosen. The theoretical sampling also included data collection from professionals that were not directly involved in care transitions between hospitals and rehabilitation but who had valuable insights in the topics of the emerging theory, i.e. registered nurse at the out-patient stroke unit at hospital and primary care physician.

Data from both hospital A and B was collected in the same way. The semi-structured individual interviews and focus group interviews were conducted by SL, MF and CY, digitally recorded and transcribed verbatim. The individual interviews and focus groups lasted between 12–70 minutes. The questions in the interview guide aimed at eliciting a description of the process of discharge from the hospital to the home and to capture the views and perspectives of the participants. The initial question referred to the decision to discharge: *“Could you please tell me about what happened when you first found out that you were going to be discharged from the stroke unit?”, “Could you please tell me about the time you first heard that your significant other was about to be discharged from the hospital?”*, and *“Could you please tell me what happens when the decision about the patient’s discharge is made?”*. The initial questions were followed by questions about what happened during the time after the decision to discharge was made and the perspective of the transition process on coming home from hospital. Probes and follow-up questions were used to get a deeper understanding of the transition process. Along with the analysis of the interviews, the interview guide was revised, when needed, to further form the data collection and explore upcoming topics or events in more depth. The focus groups were conducted with the healthcare professionals either at a hospital unit or neuro-rehabilitation unit and the number of participants varied from three to five. To adjust to the clinical environment and to avoid any unnecessary workload, interviews with healthcare professionals were sometimes carried out individually. Interviews with people with stroke and significant others, conducted concurrently with the focus group interviews, took place either individually or in dyads in participant’s home or at any other convenient location chosen by the participant.

The analytic work was performed continuously, side by side with the process of data collection, in order to form and adjust the data collection. The analysis was carried out according to the following analytic steps of grounded theory: 1) Initial coding; 2) Focused coding; 3) Axial coding; and 4) Theoretical coding [23]. Transcriptions were initially coded by SL and MF. CY reviewed and contributed to this iterative and interactive process. All the researchers agreed on the initial codes and emerging categories and discussed ongoing data collection and analysis. Throughout the analysis, a constant comparative method was used in order to analyse and compare data within and between interviews and to confirm similarities and differences. In order to stay close to the data and create an understanding of the transition process, initial coding was performed with line-by-line coding of each interview. After the initial coding a condensation of existing codes to categories was performed, to find similarities and differences in data, to form and adjust the continued data collection. After condensation and categorizing of the initial codes a focused coding was performed to synthesize and explain larger segments of data. The initial codes and the emerging categories were then used to form the continued data collection with the intention to check, refine and affirm the findings. As a next step of the analysis, axial coding was performed in order to relate categories to subcategories on a conceptual level. After axial coding a process of theoretical coding with a conceptualization of categories took place. In this process relationships between categories were identified and integrated to a core category.

## Results

**Figure 1:**
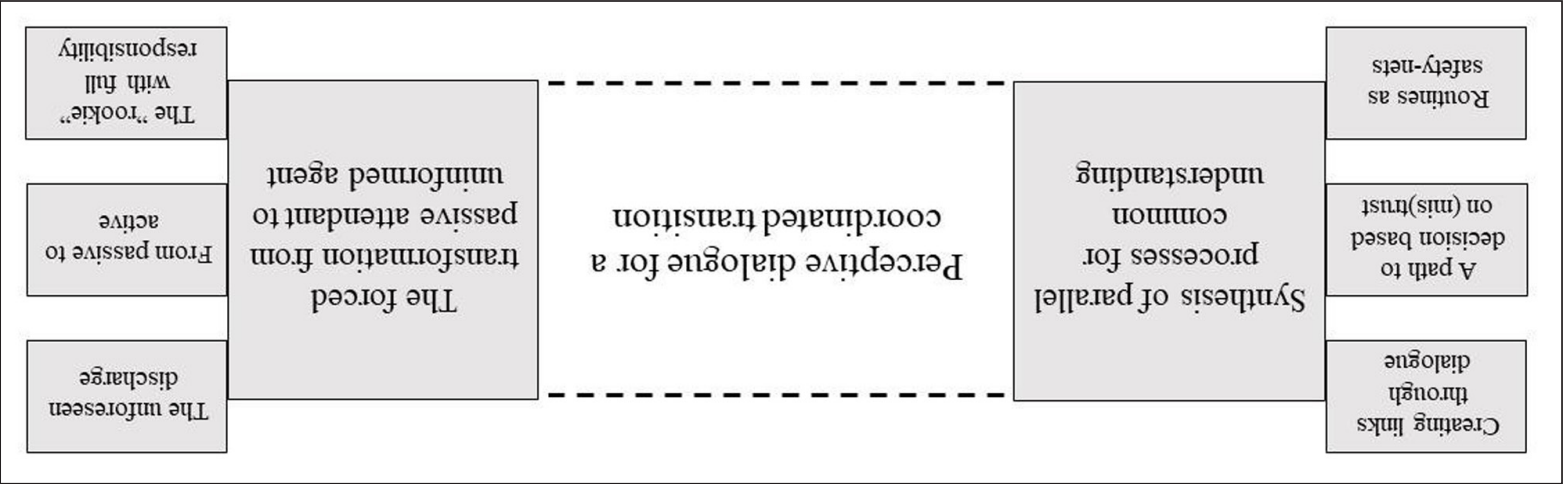
Core category, categories and subcategories in the result.

One core category *“Perceptive dialogue for a coordinated transition”*, and two categories “*Synthesis of parallel processes for common understanding”* and *“The forced transformation from passive attendant to uninformed agent”*, each with three subcategories resulted from the analysis, see Figure 1. The core category *“Perceptive dialogue for a coordinated transition”* reflects the emerging theory. A perceptive dialogue for a coordinated transition entails the need for responsiveness for each other and each other’s specific situation and context during the transition – both from the perspective of patients, significant others and healthcare professionals. This may both depend on and lead to a shared trust, routines and mutual involvement of all in dialogues. A perceptive dialogue facilitates the synthesis of processes that form a common understanding of the transition as a whole and partnership between patients and professionals. On the other hand, a lack of perceptive dialogue between patients and professionals’ risks leading to an unforeseen transition where patients are forced to a sudden responsibility.

### Synthesis of parallel processes for common understanding

The transition process was made up of several parallel processes consisting of individual assessments, individual or team decisions regarding subsequent care, communication of decisions to patients, provision of information to out-of-hospital caregivers, and the receiving of information of the neuro-rehabilitation teams. These parallel processes had to be synthesized for a common understanding of the patients’ needs and for making well-founded decisions about patients’ further care and rehabilitation. The synthesis of processes could be done through links, a shared trust between individuals and between organizations, and routines.

#### Creating links through dialogue

All the professionals described how a well-functioning transition was dependent on links which were achieved through meeting each other and sharing information within and between organizations. Several prerequisites for such links were identified: spatial proximity, formal and structured team meetings, and formal cross-organizational dialogue.

Spatial proximity within organizations with co-location of team members from different professions was said to facilitate dialogue. The neuro-rehabilitation teams in the primary care described how spatial proximity between team members facilitated close collaboration and enabled them to form a comprehensive understanding of the patient’s needs. Further, it facilitated dialogue concerning patients and enhanced knowledge of the other professions’ role.

One of the hospitals had formal and structured daily team meetings, which enabled direct face-to-face communication. The meetings aimed to synthesize individual assessments and actions by team members as a basis for well-founded team decisions about patients’ subsequent care. The team meetings also provided an opportunity to change or withdraw previous decisions as required by altered conditions.

On the other hand, healthcare professionals at a hospital without formal and structured daily meetings considered that the absence of such meetings led to individual physicians making decisions on subsequent care, risking the patient’s needs not being fully addressed. It also meant time-consuming efforts to communicate information to other members of the team. Information was either disseminated by means of the patients’ medical record with a risk of loss of information or through “corridor talk”, where team members exchanged information in hospital corridors if they ran into each other. The corridor talk was dependent on personal relationships, which could lead to arbitrariness as to when, how and what was communicated.

*“Instead of going to look for one another in the corridor or…It takes such a lot of extra time doing that, and then perhaps you hop over it and then just look in the notes.”* (Interview 018, occupational therapist)

The healthcare professionals both at hospitals and in neuro-rehabilitation teams described the absence of formal cross-organizational dialogue as a problem. The information consisted of one-way communication by electronic referral from hospitals to neuro-rehabilitation teams with reference to information in the medical records. There was no agreement as to what, or how information should be transferred. Instead, each individual had to find their own way. The absence of dialogue between levels of care was described as generating preconceptions, misunderstandings and mistrust about each other’s way of working. This was said to influence the information provided to patients. Some healthcare professionals had a restrained approach to informing patients about neuro-rehabilitation with the aim of not promising them too much post-hospital rehabilitation. Others provided information in a positive sense in order to make the patient feel safe during the transition process, but also to reduce the risk of patients declining contact with the neuro-rehabilitation team after discharge.

*“I hand over, I want help with something and I want an answer back. How did it work out? But I think it’s about communication to a great extent and so cut back as things are now, you don’t even have time even to meet the neuro team and the home team and hear ‘how do you work, how do you think and just what kind of patients do you want?’ So you sit and guess and then base what we know on those patients who come back. We don’t base our knowledge on those we never hear of. Maybe it works. We don’t know. We have no idea.”* (Interview 019, physiotherapist)

Feedback between organizations regarding patient outcomes after discharge was pinpointed as a tool both to develop collaboration itself and also to improve working methods and patient outcomes. With a rather abrupt end to hospitalization and without dialogue with corresponding neuro-rehabilitation teams, the hospital healthcare professionals had no idea whether their efforts were beneficial to the patient. The neuro-rehabilitation teams stressed that both feedback between the organizations and collaboration between hospitals and neuro-rehabilitation teams could improve patient care.

#### A path to decision based on (mis)trust

The healthcare professionals at hospital pointed out that common values and trust in each other’s competences were key components to the coordination and synthetization of individual assessments and to how the decisions about patients’ further care and rehabilitation were taken. The decision process was either steered hierarchically or carried out through a holistic process. In the first case, the individual physicians made the decisions and in the second, holistic process, the team reached unanimous decisions through dialogue. At workplaces that were steered hierarchically some of the healthcare professionals were not sure if their individual assessment was actually taken into account by the physician who made the final decision. In contrast, workplaces where the process was holistic meant that the members of the team were engaged in dialogue about the needs of the patient and the subsequent care planned. This provided a more comprehensive view of the patient’s needs.

*“How else should one otherwise have these contact points? It makes it so much easier and just that the patient needs all these entry points. All this knowledge is needed if it is to be good. You can’t take anything away. It’s not possible. The team, it’s the lubricant.”* (Interview 016, speech and language therapist)

#### Routines as safety-nets

Routines in terms of well-defined working methods and clearly stated areas of responsibility were described as reducing the risk of mistakes and giving all equal treatment. Routines were described as safety-nets to underpin continuity of care and to avoid patients falling between stools, i.e. different areas of responsibility.

*”Yes we really have a routine, nothing gets missed in our department.”* (Interview 001, occupational therapist)

The hospital healthcare professionals and those in the neuro-rehabilitation teams stressed that successful routines were based on staff continuity enabling professionals to develop and maintain trustful and well-functioning work routines together. Professionals in units with high employee turnover described how this led to lack of structure and routines, which contributed to mistakes and dissatisfied patients.

*“It feels as if there are a lot of such routines that have just fallen away … But then there has been a lack of routine altogether. But that was something I tried to get to be a routine. I was onto it a lot, that ‘you must give this at the start when the patient comes in so that the relatives see it if they are involved…’ ‘But for some reason it was difficult to get in this kind of routine… Now there’s a new doctor… New nurses…’ So I think it was in connection with that that it kind of disappeared… And then I left”* (Interview 020, physiotherapist)

### The forced transformation from passive attendant to uninformed agent

Patients and significant others described the care transition as a transformation from passive attendant at hospital to becoming an uninformed agent at home after discharge. Absence of active involvement and dialogue contributed to a sense of uncertainty and of lack of control. Patients considered that all the responsibility for their own care was forced upon them without support or preparation as how to manage their health on their own.

#### The unforeseen discharge

Upon discharge several patients and significant others experienced the ending of their hospital stay as stressful and even forced, accompanied by a sense of being pushed out. Some patients also pointed out that they did not have a discharge meeting, which led to a sense of uncertainty, lack of control of the situation and understanding of what would happen after discharge.

*“It was at the end when he had to go home, it was only during the last days there, when I understood that he wouldn’t be allowed to stay, even though he was so ill…And so it was that discharge which just came like a …. shot! With no support. It was messed up quite simply. It just felt like ‘get out of here, we don’t care about you any more’. It was awful.”* (Interview 023, significant other)

The forced discharge process was described as affecting patient care. One patient described how before being discharged he had to check that the professionals’ tasks were carried out, for example that they took his blood pressure. Hospital healthcare professionals described how late decisions about discharge resulting in time pressure, together with practical and administrative duties that had to be hurriedly undertaken, were reasons why patients were overlooked, and the discharge process failed.

Patients who had a prepared plan for follow-up appeared to have experienced a more satisfying home coming, while patients who did not have a clear discharge meeting or a planned follow-up left the hospital with a feeling of insecurity. Patients emphasized that a prepared discharge with the opportunity for dialogue was also favorable for the healthcare system, since it probably reduced the amount of post-discharge telephone calls to the hospital and visits to emergency care.

*“You come to a place you don’t recognize and there are a lot of people, lots of information, information that can be changed and so on. And there I think that just that kind of clarity when you are discharged, exactly as they did it, is so extremely important and valuable, that you get it with you, so that you sort of have a chance to go back and look that Oh! That was it or Oh yes! That explains why it’s this way.”* (Interview 026, patient)

#### From passive to active

Patients also experienced a lack of involvement and dialogue regarding all aspects of their care at hospital such as being informed about procedures, getting feedback on test-results or being involved in decision making.

*“I suppose the doctor came in… I believe I was there for most of the day without seeing a doctor… they must have been there at the start briefly, then there was someone who checked my heart, the results on that machine … Otherwise you just lie there and wait. But perhaps that’s the way it is in hospital…”* (Interview 027, patient)

This was in contrast to experiences of increased involvement when meeting the neuro-rehabilitation teams in the home. Patients described feeling that healthcare professionals at hospital turned on the auto-pilot when in contact with the patient. A majority wished to be informed about test-results and assessments to a greater extent than was the case. Patients experienced a lack of dialogue, with healthcare professionals who were only interested in obtaining information without providing any back to the patient.

*“Yes, perhaps they should say exactly what they are putting … testing on you. They put on electrodes and stuff here and there, and what are they for and so on? I didn’t know then because I haven’t been in hospital before. You got to know afterwards. Just when they are at those things … Then they just put them on so to speak. They don’t say anything”* (Interview 022, patient)

Significant others described how they had a passive role during the hospital stay that suddenly evolved into an active role after discharge. Both patients and significant others perceived that they were expected to self-manage at home without any preparation. They were left alone with unanswered questions and without knowing where to turn with questions. Significant others had to take the role as caregivers, but also act as coordinators between different healthcare providers. They expressed a wish to be more involved in the early stages of the care episode. They also emphasized the importance of being prepared for what would happen after discharge and informed as to how they best could support the person with stroke.

*“And yes, I think, supportive, that …yes with help and information, and sort of not this hospital information … only more ‘this is probably what it will be like’ a little like ‘remember that perhaps you need to take on this bit, here is a telephone number,’ all the human bit … Because it is pretty challenging…”* (Interview 028, significant other)

At one of the hospital units a dialogue meeting for significant others was provided. It involved all the professionals on the ward and consisted of discussion about the past, the present and the future of the patient with stroke. It was described as a tool for both informing and involving significant others as well as for gaining valuable information about the patient in order to provide and plan future care. Significant others who attended this meeting felt involved and were satisfied regarding their own role and felt in control of the situation. A physician responsible for these dialogue meetings described them as time consuming, but time saving in the long run since misunderstandings could be avoided and everyone could strive towards the same goal.

#### The “rookie” with full responsibility

Patients and significant others described feeling like rookies with full responsibility for the course of the care trajectory. Patients expressed a need for support on entering a new context as a patient with stroke.

*“No it is certainly the thing like that someone takes care of you, the situation you have landed up in is so uncertain. One day you are fully functional person and so suddenly the next day you aren’t and then you land up in hospital and then you need someone who so to speak gives you maybe a framework and tells you how you can get help.”* (Interview 008, patient)

The need for support was described in terms of guidance in coping with the illness itself and its treatment, but also with the practicalities of the healthcare system. Some of the patients and significant others, being newcomers in the healthcare context, described how not knowing what rights patients had made it difficult to know what to ask for and how to make proper demands on the healthcare system. On the other hand patients who had a previous experience of healthcare reported being better prepared for what would happen and what they as a patient needed and could ask for.

*“But as I don’t have the least idea what to ask or what to think or say … I knew nothing. Then it is also hard to know what to ask. So that … absolutely, if I had only asked it would have helped. I didn’t know what I should ask. So in the end you just … what the hell … I lean back and then it … it’ll be OK.”* (Interview 028, significant other)

Most of the patients and the significant others described the importance of having a trustful healthcare professional to care for them in order to feel secure, especially during the first period after discharge. Patients also expressed a wish to have someone to turn to with questions or worries. They also named the importance of knowing they had a planned follow-up visit, either from the neuro-rehabilitation team or the stroke-unit. Both patients and significant others described the neuro-rehabilitation team as a valuable resource when they came home, someone to turn to with question who provided a feeling of safety. The neuro-rehabilitation team described how patients often had unanswered questions. These were often of a medical nature which were difficult for the neuro-rehabilitation team to answer due to the lack of medical expertise within the team. Further they described how they often had to embrace the role of coordinator, although it sometimes meant that the efforts undertaken tended to go beyond their area of responsibility.

## Discussion

This study explored the transition process from hospital to the home with continued rehabilitation from the experiences of people with stroke, their significant others and healthcare professionals from both hospital and primary care settings. This transition consisted of several parallel processes, which made it difficult for the stakeholders to get a common understanding of the transition process as a whole. Insofar as those responsible for the coordination of the care trajectory, patients and significant others, were uninvolved this interfered with the possibility of providing a transition based on the needs, values and expectations of the individual.

Perceptive dialogue was described as necessary to synthesize the parallel processes and create a common understanding of the whole care trajectory. A perceptive dialogue contributed to exchange of information, synthetization of efforts to make well-founded decisions, and improved knowledge about and trust in each other both within, and between organizations. It was also described as a course of action aimed at forming a partnership between professionals, patients and significant others in the transition process. In the literature, dialogue has been described as an important means to address diversity and barriers between professionals when learning how to work together [25]. It has also been characterized by the exchange of perspectives, information and ideas that contribute to mutual learning, as well as a change of culture from diverse disciplinary actions to a focus on meeting patient needs [26]. Our findings emphasize that to accomplish perceptive dialogue in the care transition process both normative and functional prerequisites are needed [27]. Normative prerequisites consisted of common values within the teams and a holistic way of working. Functional prerequisites consisted of spatial proximity, and routines for interaction within teams, across team and between professionals and patients. Through this, the perceptive dialogue could be the bridge between sites – compared to ESD, in which the transition is bridged by individual professionals who follows the patient through the system.

Spatial distance between members who were placed in different organizational units lead to a relational distance and interfered with their dialogue with each other. In this study, as well as in the literature, spatial proximity, with co-location of healthcare professionals, was identified as contributing to multi-professional communication and collaboration [26,28,29,30]. Co-location has also been reported as enabling and facilitating the integration of care [31,32]. However, co-location itself does not necessarily lead to improvements in collaboration and integration of care [33] as the organizations also need to consider management support, shared visions, professional boundaries and channels for communication. Hence, to enable this, there is a need to provide forums for dialogue and collaboration in addition to the way the healthcare professionals’ work is organized. In the present study, the absence of such forums led to healthcare professionals creating informal ways of communicating or relying instead on dissemination of information by means of the medical record. Such informal communication may compensate and fill in the gaps caused by a lack of formal ways of communicating [34,35]. However, it has been shown that informal communication also entails a risk of loss of information as it only happens by chance [36]. In addition to underscoring the importance of forums for dialogue and collaboration, the absence of cross-organizational communication is a barrier to the coordination of care and continuity of information [37,38,39]. As patients need realistic information about their subsequent care, this could have a seriously negative impact on patients’ health and the provision of information, as well as hindering or delaying continued rehabilitation and follow-up procedures. In order to minimize the risk of patients getting misleading information, all communication about subsequent care must be based on facts and not on beliefs. This calls for procedures to improve integration both within and across healthcare organizations. Enabling multiple modes of communication, such as face-to-face communication and agreement about guidelines and treatment plans have been identified as facilitators for effective transitions [40]. Technology, such as video-communication tools could be used to facilitate cross-organizational communication and learning [41]. This could contribute to a common understanding of the transition process and increased trust between healthcare professionals at hospitals and the neuro-rehabilitation teams in primary care [42,43].

Irrespective of the way the hospital units organized their work and regardless of whether the way of working with patients was holistic or hierarchical, the patients and significant others felt uninvolved during the transition process. The lack of dialogue and involvement spanned across information about patient’s health, and what had been done at the hospital, to information about and preparation for self-management after discharge. Patients and significant others emphasized that their perspectives were crucial, but often neglected. The professionals in this study referred to late decisions about discharge together with their many practical and administrative duties as reasons to why transitions and involvement of patients sometimes failed. Workload, lack of time and competing interests have previously been reported as reasons for low patient involvement [44] but also that a lot of effort is put into preparing the discharge and less into the actual information provided to patients [45]. Further, our results highlight the patients’ need for support and knowledge about their condition. This is especially important for patients with stroke, who are “rookies” in healthcare, due to the sudden onset and the often continuous need of rehabilitation and self-management. The patients’ experienced lack of information about their condition, which could further explain the low level of involvement. Lack of knowledge has been shown to hinder patient participation, but it also affects the balance of power between healthcare professionals and patients [46]. Patient involvement has also been connected to increased self-efficacy [47] and better health outcomes [48].

Patients and significant others described how their discharge was unforeseen and accompanied by a feeling of being pushed out. Similar findings have been found in patients with multiple chronic illnesses [49,50]. The short length of stay in acute stroke care settings, with limited time to fully involve the patients during their hospital stay, might contribute to the experience of being pushed out. However, some patients in our study whose stay was longer also felt that their discharge was rushed. This could indicate that healthcare professionals’ actions and efforts are more important than the length of stay in relation to patient’s experiences of a forced discharge. Already involving patients and the significant others during the early phase of the hospital stay might improve their experiences and increase the healthcare professionals’ chances of providing person-centred transitions. Involving patients in their own care, and the planning of subsequent care, could hopefully enhance their knowledge about their condition and thereby increase self-efficacy and health outcomes. Preparation as to what to expect of the hospital stay could also reduce the feeling caused by a sudden discharge. It could also be argued that most information should be provided and planning of rehabilitation done post-discharge to avoid excessive information being given during the short hospital stay. However, this should not exclude awareness of the importance of involving the patient and significant other during the hospital stay. In order for patients to feel safe, it is still necessary for them to be involved and agree as to how things are done and at what stages of the care trajectory. It further assumes an agreement between organizations on what information should be provided, and when and how.

The strengths of this study include the theoretical sampling together with the multiple stakeholder perspective used to understand the transition process which resulted in rich data. The use of focus groups, individual and dyad interviews together with the multiple stakeholder perspective was a way of validating the data through triangulation of sources. Further, the credibility of our results was strengthened by our use of the iterative process and constant comparative method. This made it possible for us to refine and affirm our results and deepened our conceptual understanding. One limitation of the study is the question of transferability. The participants were recruited from two different hospitals in the context of the healthcare system in Stockholm, Sweden, which might limit the transferability of our results to other settings. As some of our findings highlight possible ways to improve transitions that are controlled and steered at a system or organizational level, the results might have been strengthened and a deeper understanding gained had the perspective of managers, decision makers and heads of organizations been involved.

## Conclusion

This study elucidates that a perceptive dialogue with patients/significant others and across organizations, are part of a coordinated and person-centred transition. There is an extensive need for increased involvement of patients and significant others regarding dialogue about health conditions, procedures at the hospital and preparation for self-management after discharge. This study suggests that, when organizational circumstances does not allow cross-organizational bridges, perceptive dialogue could serve as the linkage between units in order to coordinate the transition based on the needs of the individual.
